# Sugar-Containing Beverages Consumption and Obesity in Children Aged 4–5 Years in Spain: the INMA Study

**DOI:** 10.3390/nu11081772

**Published:** 2019-08-01

**Authors:** Sandra Gonzalez-Palacios, Eva-María Navarrete-Muñoz, Manoli García-de-la-Hera, Laura Torres-Collado, Loreto Santa-Marina, Pilar Amiano, Maria-Jose Lopez-Espinosa, Adonina Tardon, Isolina Riano-Galan, Martine Vrijheid, Jordi Sunyer, Jesus Vioque

**Affiliations:** 1 Department of Public Health, History of Medicine and Gynecology, Miguel Hernández University and Institute for Health and Biomedical Research (ISABIAL—FISABIO Foundation), 03010 Alicante, Spain; 2 Spanish Consortium for Research on Epidemiology and Public Health (CIBERESP), 28029 Madrid, Spain; 3 Subdirección de Salud Pública y Adicciones de Gipuzkoa, 20013 San Sebastian, Spain; 4 Biodonostia Health Research Institute, Basque Government, 20014 San Sebastián, Spain; 5 Foundation for the Promotion of Health and Biomedical Research in the Valencian Region, FISABIO-Public Health, 46020 Valencia, Spain; 6 University of Oviedo, 33003 Asturias, Spain; 7 Servicio de Pediatria. Hospital Universitario Central de Asturias-Oviedo, 33011 Asturias, Spain; 8 ISGlobal, Institute for Global Health, 08036 Barcelona, Spain; 9 University Pompeu Fabra, 08036 Barcelona, Spain

**Keywords:** sugar-containing beverages, obesity, packaged juices, soft drinks, preschool child

## Abstract

The consumption of sugar-containing beverages (SCB) has been associated with obesity although the evidence in preschool children is scarce. Cross-sectional analyses were performed to assess the association between obesity and SCB consumption (packaged juices and sugar-sweetened soft drinks) in 1823 children at the age of 4–5 years from the INfancia y Medio Ambiente (INMA) Project. One drink was defined as a glass of 175 mL, and the consumption of SCB was categorized in <1, 1–7 drinks/week and > 1 drink/day. We used multiple logistic regression to estimate odds ratios (OR). The average SCB consumption was 79.1 mL/day, mainly from packaged juices (80.9%). The SCB consumption was lower in non-obese children than in children with obesity, 76.6 vs 118.4 mL/day (*p* = 0.02). After adjusting for covariates, children who consumed >1 drink/day showed elevated odds of obesity, OR = 3.23 (95% confidence interval (CI): 1.48–6.98) compared to children who consumed <1 SCB drink a week. Each additional SCB drink per day was associated with higher odds of obesity, OR = 1.55 (1.14–2.09). Higher consumption of packaged juices, but not sugar-sweetened soft drinks, was significantly associated with higher odds of obesity, OR = 1.55 (1.09–2.15) and OR = 1.59 (0.76–3.39), respectively. A higher SCB consumption is associated with obesity in preschool children, mainly due to the consumption of packaged juices.

## 1. Introduction

Childhood obesity is a major public health problem not only owing to the global increase in the prevalence during the last four decades [1,2], but also to the adverse health consequences in child and adult life [3,4]. The estimated worldwide prevalence of childhood overweight and obesity in 2010 was 6.7% and is expected to reach up to 9.1% in 2020 [1]. In Europe, approximately 7% of children under the age of 10 years were estimated to have obesity according to the International Obesity Task Force (IOTF) criteria [5], similar to figures observed in Spain, where the prevalence of obesity in children from 2 to 10 years was 5.6% in boys and 6.8% in girls [6].

It has been pointed out that obesity is a complex disorder that is affected by many interacting genetic and non-genetic factors that should be further investigated [7]. In a systematic review on the causes of obesity in children that included 61 articles, it was concluded that there was sufficient evidence to support the association between low physical activity and genetic factors and the development of excessive fatness in children and adolescents, although there was less evidence for other factors such as sedentary behavior or dietary factors among which were included sugar-containing beverages (SCB) intake [8,9]. 

The SCB are non-alcoholic beverages whose main nutritional component is sugar, naturally present (e.g., fruit juices) or industrially added (e.g., fruit drinks or sugar-sweetened soft drinks). The consumption of SCB has increased in parallel with the obesity epidemic in the last few decades in the World [10] and therefore, the World Health Organization considers that it may be implicated in the obesity epidemic [11], as a probable result of the high sugar content and the low capacity of satiation of this type of drinks compared to other foods, thus providing extra calories above energy requirements and leading to weight gain [12,13,14].

The association between SCB and obesity has been extensively investigated, and despite some controversy [15,16], the present evidence supports a positive association between SCB consumption and weight gain, particularly among adults [17,18,19] and, to a lesser extent, among children [20,21,22,23,24]. In a recently published review based on 37 cohort studies carried out in children under 12 years of age, a positive association was shown between the total consumption of SCB and excess weight gain, and a positive association for fruit juices among children under 5 years of age [23]. This positive association between fruit juices and obesity in children under 5 years should be highlighted because fruit juices are the most commonly consumed SCB at this age [24]. The positive association between SCB and obesity has also been reported in children between 5 and 18 years of age from Mediterranean countries [25,26,27], although the association has not been reported by others [28,29]. Thus, it seems there is increasing evidence that the consumption of fruit juices is related to obesity in young children, although the available information is still insufficient and not fully consistent, particularly in Mediterranean areas [24].

The aim of this study was to assess the association between the consumption of SCB, including packaged juices and sugar-sweetened soft drinks, and obesity in children aged 4–5 years in Spain. 

## 2. Materials and Methods

### 2.1. Study Population and Data Collection

This is a cross-sectional analysis based on the INfancia y Medio Ambiente [Environment and Childhood]) INMA Project, a prospective population-based cohort study in 4 geographical areas in Spain: Asturias, Gipuzkoa, Sabadell and Valencia [30]. A total of 2644 women who fulfilled the inclusion criteria (≥16 years of age, intention to deliver at reference hospital, no problems of communication, singleton pregnancy and no assisted conception) were recruited during the first prenatal visit between November 2003 and February 2008. All participants provided written informed consent, and the study was approved by the Ethical Committees of the Institutions involved in the project (CEIC-Hospital La Fe, Valencia; CEIC-Hospital de Zumárraga, Gipuzkoa; CEIC-Parc de Salut Mar, Barcelona; CEIC-Hospital Universitario Central de Asturias). Excluding the women who withdrew from the study (*n* = 61), were lost from follow-up (*n* = 5), had induced or spontaneous abortions (*n* = 62), or had fetal deaths (*n* = 10), 2506 women finally delivered a live infant between May 2004 and August 2008. After birth, 1823 (72.7%) children remained in the study at the age of 4–5 years (mean 4.4 years) and provided information on the main variables. 

### 2.2. Anthropometric Measures

Weight and height were measured in children without shoes and with light clothing at the age of 4–5 years by trained staff using standard protocols. Body mass index (BMI) was estimated as weight in kilograms divided by height in meters squared (kg/m^2^), and obesity (non-obesity/obesity) was considered when girls showed a BMI of 19.12 and boys of 19.26 according to the specific cut-off proposed by the IOTF at the age of 4.5 years. [5,31] 

### 2.3. Dietary Assessment

Dietary intake of children was assessed using a semi-quantitative food frequency questionnaire (FFQ) at the time of anthropometric data collection. The FFQ was derived from an adult version that had previously been validated among the mothers of the children [32], and modified to include foods and portion sizes appropriate to children aged 4–5 years. The validity of the FFQ against three 24-hour recalls and the plasma concentration of several nutrients in a sample of 169 children aged 4–5 from the INMA-Valencia cohort was considered satisfactory [33]. The specific correlation coefficients for reproducibility were *r* = 0.62 for packaged juice and *r* = 0.50 for sugar-sweetened soft drinks.

Trained interviewers collected information from caregivers on the child’s usual diet during the previous 6–9 months for the consumption of standard units and serving size specified for each food item. The FFQ included nine possible answers to determine frequency of intake, ranking from ‘Never or <1 per month’ to ‘≥ 6 per day’. The total SCB consumption was derived from the sum of 2 questions of the FFQ on: 1) packaged or bottled juices that included major types of packaged fruit drinks and fruit juices available in Spain at the time of data collection in 2008–9 (they were basically commercial packaged fruit juices or fruit drinks with sugar added, since commercial 100% fruit juices were rarely available at that time); and, 2) sugar-sweetened soft drinks that included all types of regular soft drinks. The association with artificially-sweetened soft drinks was not analyzed in our study because of the extremely low consumption of these beverages among children at the age of 4–5 years (mean daily intake, 3.5 mL/day, approximately 0.12 oz. per day). In addition, information on “natural orange juice” (freshly prepared) was also derived from a different item of the FFQ, although the association the association between natural orange juice and obesity was not analyzed in this study since it was not considered as a SCB. One drink was defined as a glass (approximately 175 mL or 6 US fluid ounces). We calculated the usual daily nutrient intakes for each child by multiplying the frequency of the use of each food item by their nutrient content of the proportion size specified in the FFQ. Nutrient values (including total energy intake and trans-fatty acids) were obtained from the Nutrient Database for Standard Reference from the United States Department of Agriculture, Release 24 [34] and Spanish food composition tables [35].

### 2.4. Covariates

Based on previous knowledge [7], information on several variables was considered of a priori interest. Parents’ information was collected during the first and third trimester of pregnancy: mother’s age, the consumption of SCB by the mother during pregnancy, maternal educational level, maternal social class by Spanish adaptation of the International Standard Classification of Occupations system (ISCO88) [36], self-reported pre-pregnancy BMI (<25 kg/m^2^, normal weight; 25–29.99 kg/m^2^, overweight; ≥30 kg/m^2^, obesity), weight gain during pregnancy defined as the last gestational weight before delivery minus pre-pregnancy weight divided by the length of gestation in weeks at the last measurement, smoking, and self-reported paternal BMI. Additional information on child characteristics was collected: age, sex, use of formula feeding during infancy, usual intake of fruits and vegetables, water, milk, flavored milk drinks and trans-fatty acids, television viewing in hours/day and overall physical activity of the child as reported by caregivers who answered the question ‘Overall, taking in to account all the physical activity, how do you consider your child?’. Children were classified in a three-category variable as low (low-moderate), medium (fairly active) and high (active and very active). 

### 2.5. Statistical Analysis

We described the socio-demographic characteristics and lifestyle of parents and children according to obesity status (non-obesity/obesity) using mean and standard deviation for continuous variables and n and % for categorical variables. Based on the literature [7], bivariate analyses were conducted to explore potential confounding variables associated with the children’s BMI and SCB consumption, when variables showed a *p* value < 0.10, they were included in multivariable models. Three models were fitted for the presentation of the results. Model 1 adjusted for sex, age of the child and a categorical variable to account for the effect of 4-geographical areas where the study was carried out (Asturias, Gipuzkoa, Sabadell and Valencia). Model 2 adjusted for the variables in model 1 plus mother’s age, educational level and social class, weight gain during pregnancy, BMI of parents, overall physical activity, trans-fatty acids, and water and flavored milk drinks intake at 4–5 years. Model 3 adjusted for the variables in model 2 plus total energy intake. There were missing values for several variables: mother’s weight gain during pregnancy (number of missing data = 52), father’s BMI (*n* = 30) and children’s physical activity (*n* = 15). In order to maintain maximum statistical power in the analyses, we created a new category for those variables with missing values. Multiple imputation of missing values was not considered since overall, the missing values represented less than 3% of the sample. 

Models for SCB were adjusted by artificially-sweetened soft drink consumption. Models for packaged juice were adjusted by sugar-sweetened and artificially-sweetened soft drink consumption. Models for sugar-sweetened soft drink consumption were adjusted by packaged juice and artificially-sweetened soft drink consumption.

The consumption of SCB was considered as a continuous variable in units of one drink a day and as a categorical variable in 3 categories: (1) less than one drink a week; (2) 1 to 7 drinks a week; and (3) more than 1 drink a day. Therefore, children who consumed 1 drink a day are included in the category of 1 to 7 drinks a week. Packaged juice and sugar-sweetened soft drinks could not be categorized in three weekly frequencies due to low numbers in the upper categories of consumption. 

Prevalence odds ratios and confidence intervals were estimated by multivariable logistic regression in order to explore the association between obesity (non-obesity/obesity) and the total and subtypes of SCB consumption. Sensitivity analysis was conducted to evaluate the robustness of the findings using odds ratios for the consumption per one drink a day of SCB, packaged juice and sugar-sweetened soft drinks using the model 3 as a base model (*n* = 1823). Implausible dietary intakes were calculated for each participant by applying the formula proposed by Goldberg et at [37] with the cut-off points for European 2 to 6-year-old children published by Börnhorst et al. [38]. We identified 63 children under-reporters and 1 over-reporter; they were not removed from the data, but their effects on the results were explored in sensitivity analyses after their exclusion (final *n* = 1744). In addition, we performed other sensitivity analyses by excluding children with overweight from the non-obesity category (final *n* = 1556). Moreover, we further adjusted multivariate models by adding each of the following variables separately: maternal SCB consumption during pregnancy (final *n* = 1724), use of formula feeding (final *n* = 1773), milk intake (final *n* = 1823), fruit and vegetable intake (final *n* = 1823), and television viewing (final *n* = 1823) at 4–5 years. 

All the statistical analyses were performed using the program R 3.5.1 (R Foundation for Statistical Computing, Vienna, Austria; http://www.R-project.org), and *p*-values < 0.05 were considered statistically significant. 

## 3. Results

The overall prevalence of obesity was 5.9%, ranging from 3.5% in Gipuzkoa to 9.3% in Asturias. The main sociodemographic and lifestyle characteristics of the parents and children according to the non-obesity and obesity status of children are shown in Table 1. A lower maternal educational level and social class, smoking during pregnancy, and higher parental BMI and maternal weight gain were more common in children with obesity (*p* < 0.05). Moreover, children with obesity showed a higher use of formula feeding during infancy, higher level of television viewing, and intake of trans-fatty acids, and lower overall physical activity and milk intake (*p* < 0.05). 

The mean daily consumption of SCB was significantly higher (118.4 vs 76.6 mL) in children with obesity than children without (Table 2). Children with obesity also showed a higher consumption of packaged juice (*p* = 0.03) than children without obesity (Table 2). 

In the most fully adjusted multivariate models, the consumption of SCB and packaged juices were positively associated with obesity in children at the age of 4–5 years (Table 3). Compared to the consumption of less than one drink/week of SCB, the consumption of more than one drink/day was associated with higher prevalence of obesity, OR = 3.23 (IC 95% 1.48-6.98). The consumption of one drink of SCB a day was associated with a significantly higher prevalence of obesity, OR = 1.55 (IC 95% 1.14–2.09). We also observed positive significant associations for the consumption of one drink of packaged juice, OR = 1.55 (IC 95% 1.09–2.15), and for one drink of sugar-sweetened soft-drinks, OR = 1.59 (IC 95% 0.76–3.39), although this association was not statistically significant. When total energy intake was not included in the multivariate analysis, the association between the consumption of SCB, packaged juice and sugar-sweetened soft drinks, and obesity remained practically unchanged.

**Figure 1:**
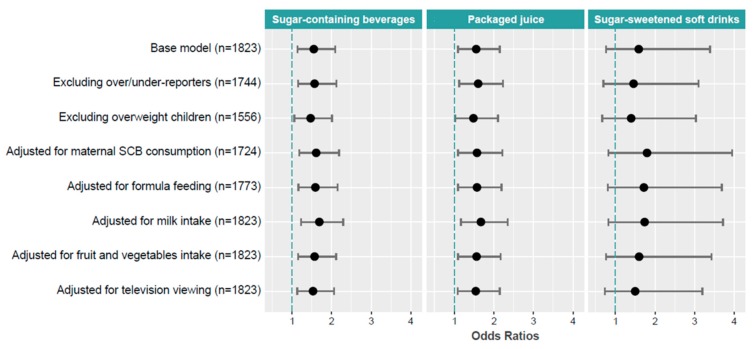
Sensitivity analyses for the association between sugar-containing beverages, packaged juice and sugar-sweetened soft drinks consumption and obesity based on model 3 of Table 3.

Figure 1 shows the results of sensitivity analyses for the association between the consumption of one drink of SCB, packaged juice and sugar-sweetened soft drinks, and obesity. The associations were practically unchanged for all the sensitivity analyses, although the associations were slightly increased when maternal SCB consumption during pregnancy was added to the multivariate analysis.

## 4. Discussion

This study suggests that the consumption of SCB is associated with obesity in children 4–5 years old. The association seems to be mainly mediated by the consumption of packaged juices, by far the most frequently SCB consumed by the children in our study (3 out 4 SCB drinks were from packaged juices). We have also observed that the consumption of sugar-sweetened soft drinks was associated with obesity although this association was not statistically significant, probably because of the lack of statistical power in part due to the much lower consumption of soft drinks among preschool children.

The direct association between SCB consumption and weight gain, overweight, and obesity in children and adolescents from 6 months to 19 years of age was assessed in an umbrella analysis of systematic literature reviews and meta-analyses published before 2015, mostly based on cross-sectional studies [39]. Although our results are in part consistent with this review, we should be cautious in making direct comparisons with other studies due to differences in the age range of the children among the studies, the SCB definition, or the criteria used to define obesity in children [40,41,42,43,44]. In one cross-sectional study with 9600 children between the ages of 4 and 5 years, the SCB intake was directly associated with BMI [45]. However, other studies carried out in older children from Mediterranean areas have shown mixed results. In one study among 856 Greek children between the ages of 4 and 7 years, the consumption of sugar-added beverages was positively associated with BMI and a greater risk of being overweight or obese [26], whereas in another study with 1675 Portuguese children between the ages of 5 and 10 years, no association was found between SCB consumption and overweight and obesity [29].

To the best of our knowledge, this is the first study reporting a positive association between the consumption of packaged juices and obesity in preschool children in a Mediterranean country. A positive association between fruit juice intake and overweight has also been reported in among children aged 1–5 years in two longitudinal studies [45,46], although not in others [47,48]. The lack of consistency among studies may be due to different reasons in part mentioned previously [49], such as the small sample size of some studies [48,50], the differences in child age range [45,46,47,48,50,51], the use of different criteria for defining obesity [45,46,47], or the discrepancies in the methods used to assess and define the fruit juices (e.g., some studies only considered 100% fruit juices) [45,46,48,50,51]. It should be noted that children in our study consumed 64 mL/day of packaged juices (including fruit juices and fruit drinks), whereas the consumption reported in other studies for 100% fruit juices in preschoolers was substantially higher, ranging from 127 to 710 mL/day [45,47,48] which may also justify inconsistencies among study results. The consumption of 100% natural orange juice was low among children of our study, it was not considered as a SCB and therefore, we did not explore the independent association with obesity. However, when we included natural orange juice within the category of total SCB, the association observed between SCB and obesity was attenuated (data not shown).

The positive association between the consumption of sugar-sweetened soft drinks and obesity in our study did not reach statistical significance which could be in part due to the lack of statistical power because of the low consumption of sugar-sweetened soft drinks in children of our study (15.1 mL/day), which was much lower than that reported in the studies by Newby (32.5 mL/day) [47] and Lim (180.4 mL/day) [43]. Although a cross-sectional study among 2-year-old children has reported a higher proportion of obesity among children that consumed one or more soft drinks a day [43], other studies in US children under 5 years of age have shown inconsistent results between soft drinks and weight gain, overweight or obesity [47,52].

Regarding possible mechanisms, it has been hypothesized that the association between SCB and obesity might be related to the high sugar content and the low capacity of satiation of this type of drink compared to other food. Therefore, their usual intake can provide extra calories above energy requirements leading to weight gain [12,13,14,53]. According to our data, the mean daily consumption of SCB was 79.1 mL, mostly from packaged juices (64 mL), thus providing nearly 155 extra kJ per day (230 kJ/day in the case of children with obesity). In this sense, it has been pointed out that small differences in energy intake could produce some weight gain on the long-term if other factors such as socio-economic status or energy expenditure remained stable [54]. However, we did not find any statistically significant difference in energy intake between children with obesity and non-obese children and therefore, we should be cautious before concluding an effect of energy intake on children obesity. When we did not adjust for total energy intake in multivariate analyses (model 2 in tables), the associations observed were similar or slightly increased, although the main conclusion of the study remain the same.

However, other mechanisms may be involved as obesity is a complex condition influenced by a wide-range of factors and, occasionally, with interactions between them [7]. In this sense, several modifiable factors such as parents’ lower socioeconomic status or educational level have been associated with higher SCB consumption [55], and also with childhood obesity [56]. Children from lower maternal social class and lower educational level showed a higher consumption of SCB, and a higher risk of obesity as well (data not shown), although the significant association between SCB consumption and obesity observed in multivariable analysis in our study was adjusted for the potential effect of these two variables. We also observed higher milk consumption in children with obesity although when milk consumption was controlled in multivariate analyses, the association between SCB and obesity was only slightly increased as shown in sensitivity analyses.

Our study has several limitations. The cross-sectional analysis may limit causal interpretations of study findings owing to potential reverse causation. However, our study was nested in a prospective mother-child cohort study and information for many variables were recorded from parents prior to the outcome at an early age of the children. In addition, we ran different sensitivity analyses to evaluate the robustness of the findings such as those analyses excluding under or over-reporting energy intake children, and the results remained essentially unchanged. Another possible limitation of our study may relate to misclassification of obesity and the inclusion of children with overweight in the reference category. We performed specific sensitivity analyses by excluding children with overweight from the reference category and the association remained significant.

A further limitation of this study may relate to the measurement errors from dietary assessment. The SCB consumption of children was reported by parents and it is possible that some miss-reporting and/or underestimation may have occurred because parents did not know well the food intake of children at the preschool centers or under caregivers. Another limitation of the dietary assessment was that it did not allow us to differentiate between subtypes of packaged juices such as 100% fruit juices, fruit juices with or without sugar added or fruit drinks. In addition, we did not collect information on the way the juices were prepared and we recognize this may be a limitation of our study. Nevertheless, the FFQ captured usual diet during the previous 6–9 months, and showed a good reproducibility for packaged fruits and sugar-sweetened soft drinks and satisfactory overall validity to assess the dietary intake, thereby making less likely a potential differential misclassification. Finally, the use of prevalence odds ratios may have overestimated associations although this potential bias should be small given the low prevalence of the outcome.

The strengths of our study include the large sample of children from a population-based mother-child cohort study in four different regions in Spain, allowing us to explore the effect of a relatively wide range of consumption for different types of SCB, and the use of high quality standardized measurements and protocols to collect all the information [30].

## 5. Conclusions

In conclusion, we have observed a positive significant association between the total consumption of sugar-containing beverages and obesity in children at the age of 4–5 years. A positive significant association was also found for the consumption of packaged juices, the SCB most frequently consumed. We did not find a significant association for sugar-sweetened soft drinks probably due to the lack of statistical power since this type of SCB was less commonly consumed in children at the age of 4 years in Spain. These results and trends in the SCB consumption should be confirmed in other prospective studies.

## Figures and Tables

**Table 1:** Characteristics of the children at 4–5 years in the INfancia y Medio Ambiente (INMA) Study according to their obesity status.

Variables ^1^	Non-Obesity ^2^	Obesity	*p* ^3^
**Parental characteristics**			
Maternal age [years]; mean (SD)	30.9 (4)	31.5 (4)	0.236
Maternal education level; No. (%)			0.047
Primary or less	359 (20.9)	33 (30.8)	
Secondary	712 (41.5)	41 (38.3)	
University	645 (37.6)	33 (30.8)	
Maternal social class; No. (%)			0.004
I/II, high	424 (24.7)	14 (13.1)	
III, medium	466 (27.2)	25 (23.4)	
IV+V, low	826 (48.1)	68 (63.6)	
Maternal pre-pregnancy BMI; No. (%)			<0.001
Normal weight	1290 (75.2)	52 (48.6)	
Overweight	311 (18.1)	32 (29.9)	
Obesity	115 (6.7)	23 (21.5)	
Maternal weight gain during pregnancy; No. (%)			<0.001
Recommended	638 (37.2)	28 (26.2)	
Lower	415 (24.2)	12 (11.2)	
Higher	615 (35.8)	64 (2.8)	
Maternal smoking during pregnancy; No. (%)			0.023
No	1184 (70.2)	62 (59.6)	
Yes	503 (29.8)	42 (40.4)	
Paternal BMI; No. (%)			<0.001
Normal weight	751 (43.8)	26 (24.3)	
Overweight	747 (43.5)	50 (46.7)	
Obesity	191 (11.1)	29 (27.1)	
**Children characteristics**			
Sex; No. (%)			0.765
Female	832 (48.5)	50 (46.7)	
Male	884 (51.5)	57 (53.3)	
Formula feeding during infancy; No. (%)			0.037
No	67 (4.0)	0 (0.0)	
Yes	1601 (96.0)	105 (100.0)	
Physical activity; No. (%)			0.028
Low active	634 (36.9)	55 (51.4)	
Moderate active	803 (46.8)	39 (36.4)	
Very active	265 (15.4)	12 (11.2)	
Television viewing [hours/day]; No. (%)			0.013
<1	517 (30.1)	17 (15.9)	
1–2	875 (51.0)	63 (58.9)	
>2	300 (17.5)	26 (24.3)	
Total energy intake [kJ/day]; mean (SD)	6598 (1452)	6879 (1665)	0.212
Trans-fatty acids intake [g/day]; mean (SD)	1.1 (0.4)	1.2 (0.5)	0.008
Water intake [mL/day]; mean (SD)	613 (280)	643 (276)	0.263
Milk intake [mL/day]; mean (SD)	321 (212)	403 (250)	<0.001
Flavored milk drinks intake [mL/day]; mean (SD)	38 (70)	33 (82)	0.806
Natural orange juice intake [mL/day]; mean (SD)	31.8 (44.8)	36.3 (43.6)	0.109
Fruits and vegetable intake [g/day]; mean (SD)	218 (122)	228 (139)	0.669

^1^ Figures for some variables may differ from the total sample (*n* = 1823) due to missing values. ^2^ Non-obesity children group includes children with normal weight and overweight. ^3^ *p*-values from Wilcoxon, Chi-squared or Fisher’s exact tests. BMI = body mass index. SD = standard deviation

**Table 2:** Mean daily consumption of sugar-containing beverages in children at 4–5 years in the INMA study according to their obesity status.

	Total *n* = 1823	Non-obesity ^1^ *n* = 1716	Obesity *n* = 107	*p* ^2^
Sugar-containing beverages ^3^ [mL/day]; mean (SD)	79.1 (109)	76.6 (103)	118.4 (179)	0.019
<1 drink/week; %	36.2	37.0	21.5	0.003
1–7 drinks/week; %	53.7	53.2	59.8	
>1 drink/day; %	10.2	9.8	18.7	
Packaged juices [mL/day]; mean (SD)	64.0 (93)	62.3 (89)	91.9 (137)	0.029
Sugar-sweetened soft drinks [mL/day]; mean (SD)	15.1 (43)	14.3 (39)	26.5 (82)	0.131

^1^ Non-obesity children group includes children with normal weight and overweight. ^2^ p-values from t-Student or chi-squared test. ^3^ Sugar-containing beverages includes packaged juices and sugar-sweetened soft drinks. SD = standard deviation; One drink = 175 mL ≈ 6 fl. oz.

**Table 3:** Associations between sugar-containing beverages consumption and obesity among children at 4–5 years in the INMA Study.

	Non-Obesity ^1^	Obesity	OR	95% CI
Sugar-containing beverages ^2,3^				
*Model 1*				
<1 drink/week	635	24	1	
1–7 drinks/week	913	65	2.05	[1.28; 3.39]
<1 drink/day	168	18	3.21	[1.65; 6.14]
Per one drink/day			1.55	[1.23; 1.93]
*Model 2*				
<1 drink/week	635	24	1	
1–7 drinks/week	913	65	1.91	[1.16; 3.24]
<1 drink/day	168	18	3.44	[1.64; 7.15]
Per one drink/day			1.58	[1.19; 2.07]
*Model 3*				
<1 drink/week	635	24	1	
1–7 drinks/week	913	65	1.88	[1.14; 3.20]
>1 drink/day	168	18	3.23	[1.48; 6.98]
Per one drink/day			1.55	[1.14; 2.09]
Packaged juices ^3^				
*Model 1*; per one drink/day			1.58	[1.18; 2.04]
*Model 2*; per one drink/day			1.58	[1.13; 2.15]
*Model 3*; per one drink/day			1.55	[1.09; 2.15]
Sugar-sweetened soft-drinks ^3^				
*Model 1*; per one drink/day			1.89	[1.11; 3.11]
*Model 2*; per one drink/day			1.58	[0.77; 3.31]
*Model 3*; per one drink/day			1.59	[0.76; 3.39]

Model 1: Adjusted for age and sex of the children (*n* = 1823). Model 2: Adjusted for variables model 1 and parental BMI, weight-gain during pregnancy, mother’s age, mother educational level, mother social class, child physical activity, child intake of *trans-*fatty acid, water and flavored milk drinks (*n* = 1823). Model 3: Adjusted for variables in model 2 plus total energy intake. (*n* = 1823). ^1^ Non-obesity children group includes children with normal weight and overweight; ^2^ Sugar-sweetened beverages include packaged juices and sugar-sweetened soft drinks. ^3^ Models for Sugar-containing beverages were adjusted by artificially-sweetened soft drink consumption. Models for packaged juices were adjusted by sugar-sweetened and artificially-sweetened soft drink consumption. Models for sugar-sweetened soft drink consumption were adjusted by packaged juice and artificially-sweetened soft drink consumption. One drink = 175 mL ≈ 6 fl. oz.
